# Non contiguous-finished genome sequence and description of *Peptoniphilus obesi* sp. nov.

**DOI:** 10.4056/sigs.3276687

**Published:** 2013-02-25

**Authors:** Ajay Kumar Mishra, Perrine Hugon, Jean-Christophe Lagier, Thi-Thien Nguyen, Catherine Robert, Carine Couderc, Didier Raoult, Pierre-Edouard Fournier

**Affiliations:** 1 Aix-Marseille Université, Marseille, France

**Keywords:** *Peptoniphilus obesi*, genome

## Abstract

*Peptoniphilus obesi* strain ph1^T^ sp. nov., is the type strain of *P. obesi* sp. nov., a new species within the genus *Peptoniphilus*. This strain, whose genome is described here, was isolated from the fecal flora of a 26-year-old woman suffering from morbid obesity. *P. obesi* strain ph1^T^ is a Gram-positive, obligate anaerobic coccus. Here we describe the features of this organism, together with the complete genome sequence and annotation. The 1,774,150 bp long genome (1 chromosome but no plasmid) contains 1,689 protein-coding and 29 RNA genes, including 5 rRNA genes.

## Introduction

*Peptoniphilus obesi* strain ph1^T^ (=CSUR=P187, =DSM =25489) is the type strain of *P*. *obesi* sp. nov. This bacterium is a Gram-positive, anaerobic, indole-negative coccus that was isolated from the stool of a 26-year-old woman suffering from morbid obesity and is part of a study aiming at cultivating all species within human feces, individually [1].

Widespread use of gene sequencing, notably 16SrRNA, for the identification of bacteria recovered from clinical specimens, has enabled the description of a great number of bacterial species and genera of clinical importance [2,3]. The recent development of high throughput genome sequencing and mass spectrometric analyses has provided unprecedented access to a wealth of genetic and proteomic information [4].

The current classification of prokaryotes, known as polyphasic taxonomy, relies on a combination of phenotypic and genotypic characteristics [5]. However, as more than 3,000 bacterial genomes have been sequenced [6] and the cost of genomic sequencing is decreasing, we recently proposed to integrate genomic information in addition to their main phenotypic characteristics (habitat, Gram-stain reaction, culture and metabolic characteristics, and when applicable, pathogenicity) in the description of new bacterial species [7-18].

The commensal microbiota of humans and animals consists, in part, of many Gram-positive anaerobic cocci. These bacteria are also commonly associated with a variety of human infections [19]. Extensive taxonomic changes have occurred among this group of bacteria, especially in clinically-important genera such as *Finegoldia*, *Parvimonas*, and *Peptostreptococcus* [20]. Members of genus *Peptostreptococcus* were divided into three new genera, *Peptoniphilus*, *Anaerococcus* and *Gallicola* by Ezaki [20]. The genus *Peptoniphilus* currently contains eight species that produce butyrate, are non-saccharolytic and use peptone and amino acids as major energy sources: *P. asaccharolyticus*, *P. harei*, *P. indolicus*, *P. ivorii*, *P. lacrimalis* [20], *P. gorbachii*, *P. olsenii*, and *P. methioninivorax* [21,22].

Members of the genus *Peptoniphilus* have been isolated mainly from various human clinical specimens such as vaginal discharges, ovarian, peritoneal, sacral and lachrymal gland abscesses [23]. In addition, *P. indolicus* causes summer mastitis in cattle [23].

Here we present a summary classification and a set of features for *P. obesi* sp. nov. strain ph1^T^ (CSUR=P187, DSM=25489) together with the description of the complete genomic sequence and its annotation. These characteristics support the circumscription of the species *P. obesi*.

## Classification and features

A stool sample was collected from a 26-year-old woman living in Marseille (France), who suffered from morbid obesity: BMI = 48.2 (118.8 kg, 1.57 meter). At the time of stool sample collection, she was not a drug-user and was not on a diet. The patient gave an informed and signed consent, and the agreement of local ethics committee of the IFR48 (Marseille, France) were obtained under agreement 09-022. The fecal specimen was preserved at -80°C after collection. Strain ph1^T^ (Table 1) was isolated in 2011 by anaerobic cultivation on 5% sheep blood-enriched Columbia agar (BioMerieux, Marcy l’Etoile, France) after 26 days of preincubation of the stool sample in an anaerobic blood culture bottle enriched with sterile blood and rumen fluid.

**Table 1:** Classification and general features of *Peptoniphilus obesi* strain ph1^T^ according to the MIGS recommendations [24]

**MIGS ID**	**Property**	**Term**	**Evidence code^a^**
	Current classification	Domain *Bacteria*	TAS [25]
		Phylum *Firmicutes*	TAS [26-28]
		Class *Clostridia*	TAS [29,30]
		Order *Clostridiales*	TAS [31,32]
		Family *Clostridiales* family XI *Incertae sedis*	TAS [33]
		Genus *Peptoniphilus*	TAS [20]
		Species *Peptoniphilus obesi*	IDA
		Type strain ph1^T^	IDA
	Gram stain	positive	IDA
	Cell shape	coccus	IDA
	Motility	nonmotile	IDA
	Sporulation	nonsporulating	IDA
	Temperature range	mesophilic	IDA
	Optimum temperature	37°C	IDA
MIGS-6.3	Salinity	unknown	IDA
MIGS-22	Oxygen requirement	anaerobic	IDA
	Carbon source	unknown	
	Energy source	peptones	NAS
MIGS-6	Habitat	human gut	IDA
MIGS-15	Biotic relationship	free living	IDA
MIGS-14	Pathogenicity Biosafety level Isolation	unknown 2 human feces	
MIGS-4	Geographic location	France	IDA
MIGS-5	Sample collection time	January 2011	IDA
MIGS-4.1	Latitude	43.296482	IDA
MIGS-4.1	Longitude	5.36978	IDA
MIGS-4.3	Depth	surface	IDA
MIGS-4.4	Altitude	0 m above sea level	IDA

Evidence codes – IDA: Inferred from Direct Assay; TAS: Traceable Author Statement (i.e., a direct report exists in the literature); NAS: Non-traceable Author Statement (i.e., not directly observed for the living, isolated sample, but based on a generally accepted property for the species, or anecdotal evidence). These evidence codes are from the Gene Ontology project [20]. If the evidence is IDA, then the property was directly observed for a live isolate by one of the authors or an expert mentioned in the acknowledgements.

**Figure 1:**
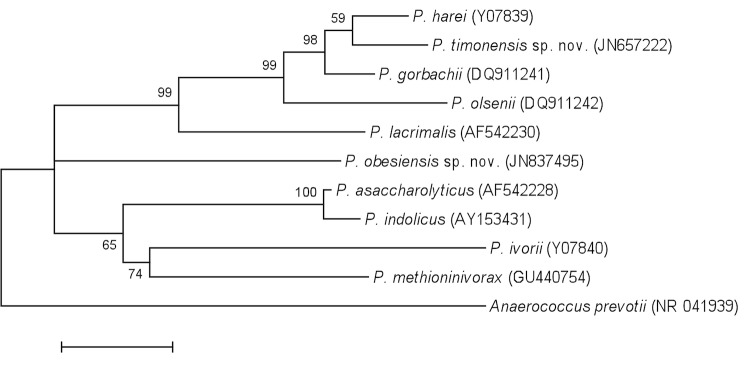
Phylogenetic tree highlighting the position of *Peptoniphilus obesi* strain ph1^T^ relative to a selection of type strains of validly published species of *Peptoniphilus*. GenBank accession numbers are indicated in parentheses. Sequences were aligned using CLUSTALW, and phylogenetic inferences obtained using the maximum-likelihood method within the MEGA software. Numbers at the nodes are percentages of bootstrap values obtained by repeating the analysis 500 times to generate a majority consensus tree. *Peptoniphilus timonensis* sp. nov., a new species that we recently proposed, was also included in the analysis [12]. *Anaerococcus prevotii* was used as outgroup. The scale bar represents a 2% nucleotide sequence divergence.

This strain exhibited a 91.0% nucleotide sequence similarity with *P. asaccharolyticus* and *P. indolicus*, the phylogenetically closest validated *Peptoniphilus* species (Figure 1). Among the validly published *Peptoniphilus* species, the percentage of 16S rRNA sequence similarity ranges from 86.0% (*P. ivoriivs. P. olsenii*) to 98.5% (*P. asaccharolyticus vs. P. indolicus*). Despite the fact that strain ph1 exhibited a 16SrRNA sequence similarity lower than the 95.0% cutoff, which is usually regarded as a threshold for the creation of new genus [2], we considered it as a new species within the *Peptoniphilus* genus.

**Figure 2:**
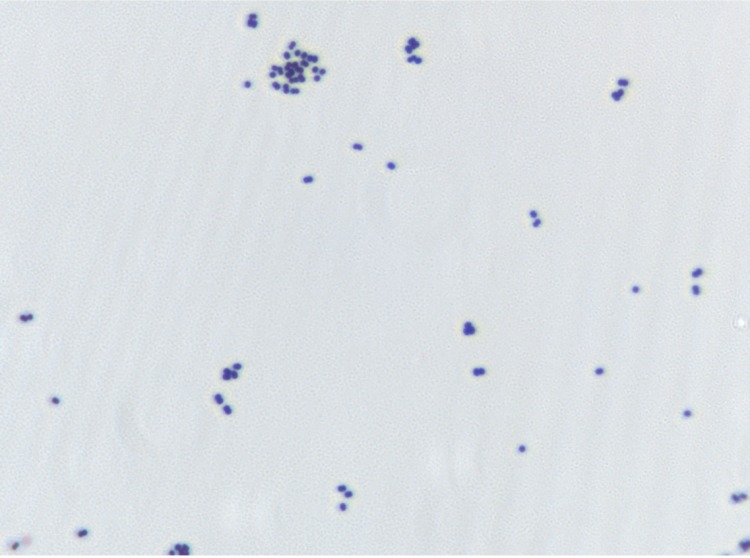
Gram staining of *P. obesi* strain ph1^T^

**Figure 3:**
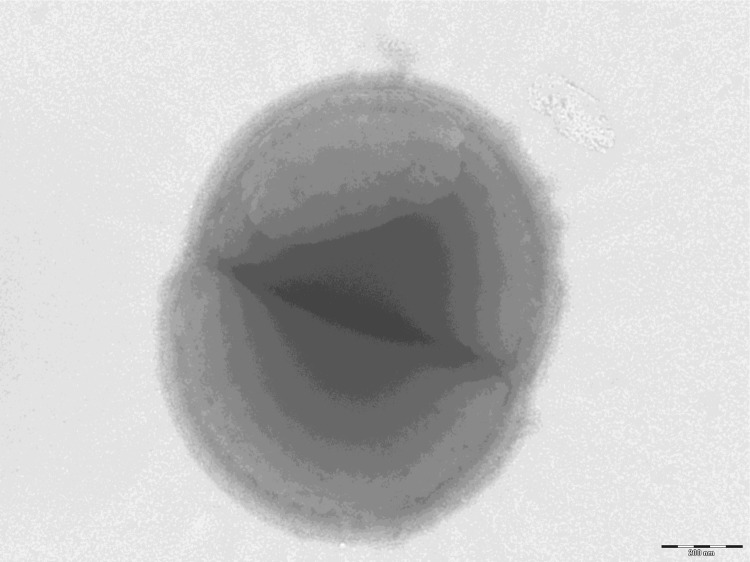
Transmission electron microscopy of *P. obesi* strain ph1^T^, using a Morgani 268D (Philips) at an operating voltage of 60kV. The scale bar represents 200 nm.

Different growth temperatures (25, 30, 37, 45°C) were tested. Growth was observed between 30°C and 45°C, with optimal growth at 37°C. Colonies stained gray, transparent, opaque, non-bright and were 0.4 mm in diameter on blood-enriched Columbia agar. Growth of the strain was tested under anaerobic and microaerophilic conditions using GENbag anaer and GENbag microaer systems, respectively (BioMérieux), and in the presence of air, with or without 5% CO_2_. Optimal growth was achieved anaerobically, but no growth occurred in microaerophilic or aerobic conditions. A motility test was negative. Cells grown on agar are Gram-positive (Figure 2) and diameter ranged from 0.77µm to 0.93 µm with a mean diameter of 0.87 µm by electron microscopy (Figure 3).

Strain ph1^T^ exhibited neither catalase nor oxidase activities. Using the API rapid ID 32A system (BioMérieux), positive reactions were observed for arginine arylamidase and leucine arylamidase. Negative reactions were found for urease, nitrate reduction, arginine dihydrolase, indole production, α-arabinosidase, α-glucosidase, α-fucosidase, β-galactosidase, glutamic acid decarboxylase, 6-phospho-β-galactosidase β-glucosidase, β-glucuronidase, N-acetyl-β-glucosaminidase, D-mannose, D-raffinose, alkaline phosphatase, alanine arylamidase, glutamyl glutamic acid arylamidase, glycine arylamidase, histidine arylamidase, leucyl glycine arylamidase, phenylalanine arylamidase, proline arylamidase, pyroglutamic acid arylamidase, serine arylamidase and tyrosine arylamidase. *P. obesi* is susceptible to penicillin G, amoxicillin, amoxicillin + clavulanic acid, imipenem, nitrofurantoin, erythromycin, doxycyclin, rifampicine, vancomycin, gentamicin 500, metronidazole and resistant to ceftriaxon, ciprofloxacin, gentamicin 10 and trimetoprim + sulfamethoxazole.

When compared with *Peptoniphilus grossensis* strain ph5^T^, *P. obesi* sp. nov strain ph1^T^ exhibited phenotypic differences as no endospore formation, no indole, no tyrosine arylamidase, no histidine arylamidase production and this strain did not fermented D-mannose. *P. obesi* sp. nov strain ph1^T^ differed from *Peptoniphilus timonensis* strain JC401^T^ by endospore formation, catalase, indole, α-galactosidase, leucine arylamidase, tyrosine arylamidase, histidine arylamidase and serine arylamidase production. *P. obesi* sp. nov strain ph1^T^ differed from *Peptoniphilus gorbachii* strain WAL 10418 ^T^ by glutamyl glutamic acid, phenylalanine arylamidase, tyrosine arylamidase and glycine arylamidase production (Table 2).

**Table 2:** Differential characteristics of *P. obesi* sp. nov strain ph1^T^, *Peptoniphilus grossensis* strain ph5 ^T^, *Peptoniphilus timonensis* strain JC401^T^ and *Peptoniphilus gorbachii* WAL 10418^T^.

**Properties**	*P.obesi*	*P.grossensis*	*P.timonensis*	*P. gorbachii*
Cell diameter (µm)	0.87	1.2	0.91	≥0.7
Oxygen requirement	anaerobic	anaerobic	anaerobic	anaerobic
Gram stain	+	+	+	+
Salt requirement	-	-	-	-
Motility	-	-	-	na
Endospore formation	-	+	+	na
**Production of**				
Phosphatase	-	-	-	-
Catalase	-	-	+	-
Oxidase	-	-	-	-
Nitrate reductase	-	-	-	-
Urease	-	-	-	-
α-galactosidase	-	-	+	-
Indole	-	+	+	var
Arginine arylamidase	+	+	+	+
Glutamyl glutamic acid arylamidase	-	-	-	+
Phenylalanine arylamidase	-	-	-	+/w
Leucine arylamidase	+	+	-	+
Tyrosine arylamidase	-	+	+	+
Alanine arylamidase	-	-	-	-
Glycine arylamidase	-	-	-	+
Histidine arylamidase	-	+	+	-
Serine arylamidase	-	-	+	-
**Utilization of**				
D-mannose	-	+	-	-
**Habitat**	human gut	human gut	human gut	human

var: variable

w: weak

na: data not available

**Figure 4:**
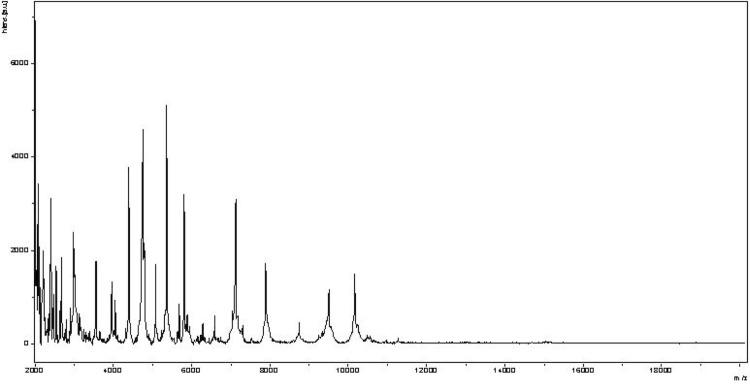
Reference mass spectrum from *P. obesi* strain ph1^T^. Spectra from 12 individual colonies were compared and a reference spectrum was generated.

**Figure 5:**
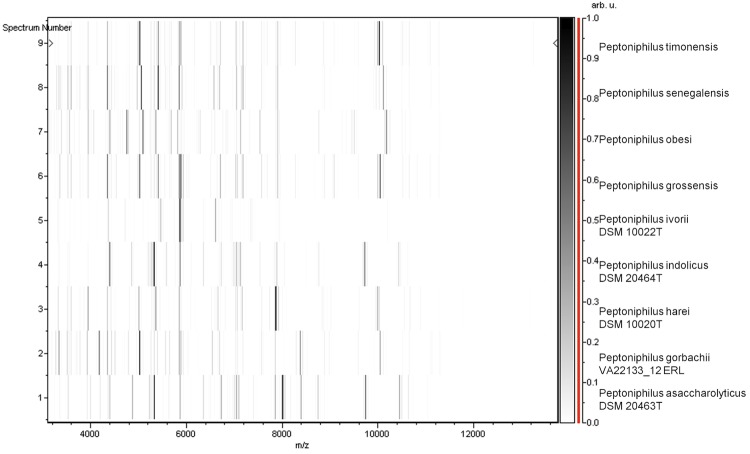
Gel view comparing *Peptoniphilus obesi* ph1^T^ spectra with other members into *Peptoniphilus* genera (*Peptoniphilus timonensis*, *Peptoniphilus senegalensis*, *Peptoniphilus grossensis*, *Peptoniphilus ivorii*, *Peptoniphilus indolicus*, *Peptoniphilus harei*, *Peptoniphilus gorbachii* and *Peptoniphilus asaccharolyticus*). The Gel View displays the raw spectra of all loaded spectrum files arranged in a pseudo-gel like look. The x-axis records the m/z value. The left y-axis displays the running spectrum number originating from subsequent spectra loading. The peak intensity is expressed by a Gray scale scheme code. The color bar and the right y-axis indicate the relation between the color a peak is displayed with and the peak intensity in arbitrary units.

Matrix-assisted laser-desorption/ionization time-of-flight (MALDI-TOF) MS protein analysis was carried out as previously described [34]. Briefly, a pipette tip was used to pick one isolated bacterial colony from a culture agar plate, and to spread it as a thin film on a MTP 384 MALDI-TOF target plate (Bruker Daltonics, Leipzig, Germany). Twelve distinct deposits were made for strain ph1^T^ from twelve isolated colonies. Each smear was overlaid with 2 µL of matrix solution (saturated solution of alpha-cyano-4-hydroxycinnamic acid) in 50% acetonitrile, 2.5% tri-fluoracetic-acid, and allowed to dry for five minutes. Measurements were performed with a Microflex spectrometer (Bruker). Spectra were recorded in the positive linear mode for the mass range of 2,000 to 20,000 Da (parameter settings: ion source 1 (IS1), 20 kV; IS2, 18.5 kV; lens, 7 kV). A spectrum was obtained after 675 shots at a variable laser power. The time of acquisition was between 30 seconds and 1 minute per spot. The twelve ph1^T^ spectra were imported into the MALDI BioTyper software (version 2.0, Bruker) and analyzed by standard pattern matching (with default parameter settings) against the main spectra of 3,769 bacteria including spectra from 8 of the 11 validly published species of *Peptoniphilus*, that are part of the reference data contained in the BioTyper database. The method of identification included the m/z from 2,000 to 20,000 Da For every spectrum, 100 peaks at most were taken into account and compared with spectra in the database. A score enabled the identification, or not, from the tested species: a score > 2 with a validly published species enabled the identification at the species level, a score > 1.7 but < 2 enabled the identification at the genus level; and a score < 1.7 did not enable any identification. For strain ph1^T^, the maximal obtained score was 1.25, thus suggesting that our isolate was not a member of a known species. We added the spectrum from strain ph1^T^ to our database for future reference (Figure 4). Finally, the gel view allows us to highlight the spectra differences with other of *Peptoniphilus* genera members (Figure 5).

## Genome sequencing and annotation

### Genome project history

The organism was selected for sequencing on the basis of its phylogenetic position and 16S rRNA similarity to other members of the genus *Peptoniphilus*, and is part of a study of the human digestive flora aiming at isolating all bacterial species within human feces. It was the seventh genome of a *Peptoniphilus* species and the first genome of *P. obesi* sp. nov. A summary of the project information is shown in Table 3. The Genbank accession number is CAHB00000000 and consists of 32 contigs arranged in 5 scaffolds. Table 3 shows the project information and its association with MIGS version 2.0 compliance.

**Table 3:** Project information

**MIGS ID**	**Property**	**Term**
MIGS-31	Finishing quality	High-quality draft
MIGS-28	Libraries used	454 GS paired-end 3-kb library
MIGS-29	Sequencing platform	454 GS FLX Titanium
MIGS-31.2	Sequencing coverage	32×
MIGS-30	Assemblers	Newbler version 2.5.3
MIGS-32	Gene calling method	PRODIGAL
	INSDC ID	PRJEA82275
	Genbank Date of Release	May 30, 2012
	NCBI project ID	CAHB00000000
MIGS-13	Project relevance	Study of the human gut microbiome

### Growth conditions and DNA isolation

*P. obesi* sp. nov. strain ph1^T^(CSUR=P187, =DSM=25489), was grown anaerobically on 5% sheep blood-enriched BHI agar at 37°C. Four petri dishes were spread and resuspended in 3x500 µl of TE buffer and stored at 80°C. Then, 500 µl of this suspension were thawed, centrifuged for 3 minutes at 10,000 rpm and resuspended in 3×100µL of G2 buffer (EZ1 DNA Tissue kit, Qiagen). A first mechanical lysis was performed by glass powder on the Fastprep-24 device (Sample Preparation system, MP Biomedicals, USA) using 2×20 seconds cycles. DNA was then treated with 2.5 µg/µL lysozyme (30 minutes at 37°C) and extracted using the BioRobot EZ1 Advanced XL (Qiagen). The DNA was then concentrated and purified using the Qiamp kit (Qiagen). The yield and the concentration was measured by the Quant-it Picogreen kit (Invitrogen) on the Genios Tecan fluorometer at 37.2 ng/µl.

### Genome sequencing and assembly

DNA (5 µg) was mechanically fragmented on a Hydroshear device (Digilab, Holliston, MA,USA) with an enrichment size of 3-4kb. DNA fragmentation was visualized through an Agilent 2100 BioAnalyzer on a DNA labchip 7500 with an optimal size of 3.287kb. The library was constructed according to the 454 GS FLX Titanium paired-end protocol. Circularization and nebulization were performed and generated a pattern with an optimum at 665 bp. After PCR amplification through 15 cycles followed by double size selection, the single stranded paired end library was then quantified on the Quant-it Ribogreen kit (Invitrogen) on the Genios Tecan fluorometer at 72 pg/µL. The library concentration equivalence was calculated as 1.99E+08 molecules/µL. The library was stored at -20°C until further use.

The shotgun library was clonally amplified with 0.5 cpb and 1 cpb in 2 SV-emPCR reactions per condition, with the GS Titanium SV emPCR Kit (Lib-L) v2 (Roche). The yield of the emPCR was 9.2% for 0.5 cpb and 12% for 1 cpb in the range of 5 to 20% from the Roche procedure. Approximately 790,000 beads were loaded on 1/4 region of a GS Titanium PicoTiterPlate PTP Kit 70×75 and sequenced with the GS FLX Titanium Sequencing Kit XLR70 (Roche). The run was performed overnight and then analyzed on the cluster through the gsRunBrowser and Newbler assembler (Roche). A total of 228,882 passed filter wells were obtained and generated 76.8Mb of DNA sequence with a average length of 336 bp. The global passed filter sequences were assembled using Newbler with 90% identity and 40 bp as overlap. The final assembly identified 5 scaffolds and 32 large contigs (>1,500 bp) generating a genome size of 1.7 Mb.

### Genome annotation

Open Reading Frames (ORFs) were predicted using Prodigal [35] with default parameters but the predicted ORFs were excluded if they spanned a sequencing gap region. The predicted bacterial protein sequences were searched against the GenBank database [36] and the Clusters of Orthologous Groups (COG) databases using BLASTP. The tRNAScanSE tool [37] was used to find tRNA genes, whereas ribosomal RNAs were found by using RNAmmer [38] and BLASTN against the GenBank database. Signal peptides and numbers of transmembrane helices were predicted using SignalP [39] and TMHMM [40], respectively. ORFans were identified if their BLASTP *E*-value was lower than 1e-03 for alignment length greater than 80 amino acids. If alignment lengths were smaller than 80 amino acids, we used an *E*-value of 1e-05. To estimate the mean level of nucleotide sequence similarity at the genome level between *Peptoniphilus obesi* and other members of the *Peptoniphilus* genera, we compared genomes two by two and determined the mean percentage of nucleotide sequence identity among orthologous ORFs using BLASTn Orthologous genes were detected using the Proteinortho software [41].

## Genome properties

**Figure 6:**
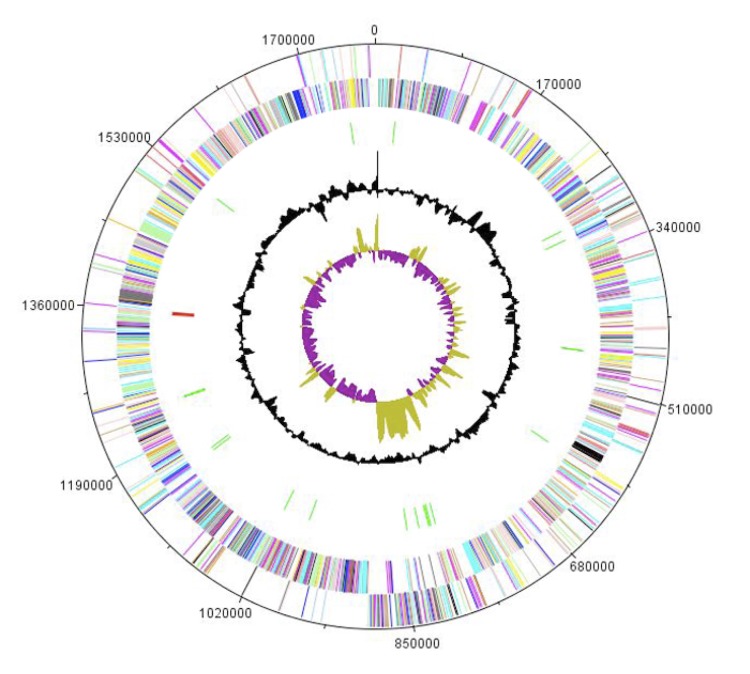
Graphical circular map of the chromosome. From the outside in, the outer two circles show open reading frames oriented in the forward (colored by COG categories) and reverse (colored by COG categories) directions, respectively. The third circle marks the rRNA gene operon (red) and tRNA genes (green). The fourth circle shows the G+C% content plot. The inner-most circle shows GC skew, purple indicating negative values whereas olive for positive values.

The genome is 1,774,150 bp long (1 chromosome, but no plasmid) with a 30.10% G+C content (Table 4 and Figure 6). Of the 1,718 predicted genes, 1,689 were protein-coding genes and 29 were RNAs. A total of 1,278 genes (74.39%) were assigned a putative function. ORFans represented 4.9% (84 genes) of the predicted genes. The remaining genes were annotated as hypothetical proteins. The distribution of genes into COGs functional categories is presented in Table 5 and Figure 6. The properties and the statistics of the genome are summarized in Tables 4 and 5.

**Table 4:** Nucleotide content and gene count levels of the genome

**Attribute**	**Value**	**% of total^a^**
Genome size (bp)	1,774,150	
DNA coding region (bp)	1,606,668	90.56
G+C content (bp)	534,019	30.1
Number of replicons	1	
Extrachromosomal elements	0	
Total genes	1,718	100
RNA genes	29	1.69
rRNA operons	1	
Protein-coding genes	1,689	98.31
Genes with function prediction	1,249	72.70
Genes assigned to COGs	1,278	74.39
Genes with peptide signals	87	5.06
Genes with transmembrane helices	414	24.10

^a^ The total is based on either the size of the genome in base pairs or the total number of protein coding genes in the annotated genome

**Table 5:** Number of genes associated with the 25 general COG functional categories

**Code**	**Value**	**% age **^a^	**Description**
J	142	8.41	Translation
A	0	0	RNA processing and modification
K	93	5.51	Transcription
L	109	6.45	Replication, recombination and repair
B	1	0.06	Chromatin structure and dynamics
D	16	0.95	Cell cycle control, mitosis and meiosis
Y	0	0	Nuclear structure
V	64	3.79	Defense mechanisms
T	43	2.55	Signal transduction mechanisms
M	48	2.84	Cell wall/membrane biogenesis
N	4	0.24	Cell motility
Z	0	0	Cytoskeleton
W	0	0	Extracellular structures
U	25	1.48	Intracellular trafficking and secretion
O	52	3.08	Posttranslational modification, protein turnover, chaperones
C	76	4.50	Energy production and conversion
G	39	2.31	Carbohydrate transport and metabolism
E	116	6.87	Amino acid transport and metabolism
F	50	2.96	Nucleotide transport and metabolism
H	46	2.72	Coenzyme transport and metabolism
I	45	2.66	Lipid transport and metabolism
P	75	4.44	Inorganic ion transport and metabolism
Q	25	1.48	Secondary metabolites biosynthesis, transport and catabolism
R	180	10.66	General function prediction only
S	129	7.64	Function unknown
-	311	18.41	Not in COGs

^a^ The total is based on the total number of protein coding genes in the annotated genome

## Comparison with the genomes from other *Peptoniphilus* species

Here, we compared the genome sequence of *P. obesi* strain ph1^T^ with those of *P. harei* strain ACS-146-V-Sch2b, *P. lacrimalis* strain 315-B, *Peptoniphilus senegalensis* JC140^T^, *Peptoniphilus timonensis* JC401^T^, *Peptoniphilus grossensis* ph5 ^T^ and *Peptoniphilus indolicus* strain ATCC BAA-1640.

The draft genome sequence of *P. obesi* strain ph1^T^ has a larger size than that of *P. lacrimalis* (1.69Mb) and *P.timonensis* (1.76Mb), but a smaller size than that of *P. harei* (1.83Mb), *P. grossensis* (2.10Mb), *P. senegalensis* (1.84Mb) and *P. indolicus* (2.20Mb). The G+C content of *P. obesi* is comparable to that of *P. lacrimalis* and *P. timonensis* (30.10%, 29.91% and 30.70% respectively) but less than that of *P. harei* (34.44%), *P. grossensis* (33.90%), *P. senegalensis* (32.20%) and P. indolicus (32.29%) *P. obesi* has more predicted ORFs than *P. lacrimalis,* (1,718 *vs* 1,586) but fewer than *P. harei, P. senegalensis, P. timonensis, P. grossensis* and *P. indolicus* (1,725, 1744, 1922, 2041 and 2262, respectively). In addition, *P. obesi* shared 931, 957, 967, 1019, 1055, 1077 orthologous genes with *P. indolicus*, *P. timonensis, P. lacrimalis, P. senegalensis, P. harei* and *P. grossensis*, respectively. The average nucleotide sequence identity ranged from 69,14% to 87,28% among *Peptoniphilus* species, and from 71,04 to 71.80% between *P. obesi* and other species, thus confirming its new species status. Table 6 summarizes the numbers of orthologous genes and the average percentage of nucleotide sequence identity between the different genomes studied.

**Table 6:** Number of orthologous genes (upper right) and average nucleotide identity levels (lower left) between pairs of genomes determined using the Proteinortho software [41].

	*P. grossensis*	*P. harei*	*P. indolicus*	*P. lacrimalis*	*P. obesi*	*P. senegalensis*	*P. timonensis*
*P. grossensis*	X	1,357	1,086	1,106	1,077	1,335	1,237
*P.harei*	82.20	X	1,078	1,095	1,055	1,297	1,195
*P.indolicus*	69.26	69.14	X	942	931	1061	977
*P. lacrimalis*	72.47	72	70	X	967	1,045	976
*P. obesi*	71.65	71.48	71.18	71.80	X	1,019	957
*P. senegalensis*	87.28	81.80	69.93	72.28	71.39	X	1,176
*P.timonensis*	82.27	83.78	70.01	72.79	71.04	82.34	X

## Conclusion

On the basis of phenotypic (Table 2), phylogenetic and genomic analyses (Table 6), we formally propose the creation of *Peptoniphilus obesi* sp. nov. that contains the strain ph1^T^. This strain has been found in Marseille, France.

### Description of *Peptoniphilus obesi* sp. nov.

*Peptoniphilus obesi* (o.be.si. L. masc. gen. adj. *obesi* of an obese, the disease presented by the patient from whom the type strain ph1^T^ was isolated).

Colonies are 0.4 mm in diameter on blood-enriched Columbia agar and stain gray, transparent, opaque, colonies are not bright. Cells are coccoid, diameter range from 0.77µm to 0.93 µm with a mean diameter of 0.87 μm.Optimal growth is achieved anaerobically. No growth is observed in aerobic conditions. Growth occurs between 30-45°C, with optimal growth observed at 37°C, on blood-enriched Columbia agar. Cells stain Gram-positive, are non endospore-forming, and non-motile. Arginine arylamidase and leucine arylamidase activities are present. Cells are negative for the following activities: catalase, oxidase, urease, nitrate reduction, arginine dihydrolase, indole production, α-arabinosidase, α-glucosidase, α-fucosidase, β-galactosidase, glutamic acid decarboxylase, 6-phospho-β-galactosidase β-glucosidase, β-glucuronidase, N-acetyl-β-glucosaminidase, D-mannose, D-raffinose, alkaline phosphatase, alanine arylamidase, glutamyl glutamic acid arylamidase, glycine arylamidase, histidine arylamidase, leucyl glycine arylamidase, phenylalanine arylamidase, proline arylamidase, pyroglutamic acid arylamidase, serine arylamidase and tyrosine arylamidase. Cells are susceptible to penicillin G, amoxicillin, amoxicillin + clavulanic acid, imipenem, nitrofurantoin, erythromycin, doxycycline, rifampicine, vancomycin, gentamicin 500, metronidazole and resistant to ceftriaxone, gentamicin 10, ciprofloxacin and trimethoprim + sulfamethoxazole.

The G+C content of the genome is 30.1%. The 16S rRNA and genome sequences are deposited in GenBank under accession numbers CAHB00000000 and JN837495, respectively. The type strain ph1^T^ (= CSUR P187 = DSM 25489) was isolated from the fecal flora of an obese French patient.
